# Editorial: Approaches to Language: Data, Theory, and Explanation

**DOI:** 10.3389/fpsyg.2020.576244

**Published:** 2020-10-22

**Authors:** Ángel J. Gallego, Aritz Irurtzun

**Affiliations:** ^1^Department of Spanish Philology, Autonomous University of Barcelona, Barcelona, Spain; ^2^CNRS, IKER (UMR 5478), Bayonne, France

**Keywords:** language, data, theory, description, analysis

This Research Topic serves as a showroom for the latest developments in linguistic methods and approaches. In so doing, the articles go beyond developing a specific research problem and they also serve as a sample of the kind of methods employed in different approaches to language, in the hope that this discussion prompts a reflection on the relation between theory, data, evidence, and explanation.

Madariaga's article is a clear vindication of the role of different factors shaping languages. It takes an I-language perspective in order to explain certain phenomena that are otherwise unapproachable such as the variation in object case marking of several Russian verbs.

Ezeizabarrena and Garcia Fernandez analyze the feasibility and utility of words or morphemes as measures for (morpho-)syntactic development in agglutinative languages such as Basque, confirming their reliability for identifying developmental patterns.

Theodorou et al. provide a pioneering analysis of sentence repetition tasks as useful tools for assessing children's language ability in bilectal settings. The study validates the diagnostic accuracy of the task, showing that it has the potential to be used as a referral criterion to identify children with Specific Language Impairment (SLI).

Leivada et al. advance in the demarcation of the linguistic phenotype of three developmental disorders: SLI, Down syndrome, and autism spectrum disorder. They perform a systematic and cross-linguistic review of their linguistic profiles and formulate the Locus Preservation Hypothesis, suggesting that aspects of the language faculty are immune to impairment across developmental disorders.

Various experimental studies address the processing of long-distance dependencies. Santesteban et al. study whether antecedent-clitic dependencies in Spanish are computed like agreement or like pronominal dependencies. They report two experiments arguing for cue-retrieval accounts of dependency resolution and suggesting that the sensitivity to attraction effects shown by clitics resembles more the computation of pronominal dependencies than that of agreement. Likewise, Sauermann and Gagarina report a visual world eye-tracking study investigating the impact of the word order and grammatical role parallelism on the online comprehension of pronouns in German. It provides compelling evidence that pronouns may not in general be associated with the subject or topic of a sentence but that their resolution is modulated by additional factors.

In a different setting, Pablos et al. present some experiments on the processing of long-distance backward dependencies in Dutch and the processing of *in-situ* wh-questions in Mandarin vs. French. This is also a study that provides a general reflection on the challenges that experimental work faces in finding a compromise between addressing theoretically relevant questions and being able to implement them in a controlled experimental paradigm.

On the more theoretical prism, Medeiros centers on design properties of language, proposing a Universal Linear Transduction Reactive Automaton (ULTRA) directly mapping surface word orders to underlying base structure with a stack-sorting algorithm.

Taking a historical and epistemological stance, Ott asks for a paced consideration of the implications of “strong generativity” in the field, and its relation to data, judgments, and their relationship with theoretical evidence.

D'Alessandro and van Oostendorp provide an even bigger picture by addressing directly broad ontological questions about our object of study and epistemological questions about how to best study it. The position that they defend is a plural one, vindicating the necessity of different disciplines, views and methodologies when studying language.

Quer and Steinbach analyze the impact of modality on linguistic data elicitation and collection, corpus studies, and experimental studies highlighting a set of specific challenges for sign language research. This paper also vindicates the complementarity of theoretical approaches and experimental studies.

Duguine proposes a new model for null subjects, and focuses on its implications for language development. The paper explores the consequences of an inverse approach to pro-drop in the domain of language acquisition, arguing that it allows to account for a number of properties of child languages.

Do and Kaiser analyze syntactic satiation effects. Their experimental analysis of Subject island and Complex-NP Constraint violations uncovers different factors that may bring about satiation, and the overall conclusion is that satiation may not be a one-size-fit-all phenomenon for different types of structures.

Bader centers on the processing of center embedding constructions in German. As a result of the discussion of the three novel experiments he reports, he argues for a multifactorial account of the limitations on center embedding in natural languages.

Giannakidou and Etxeberria review a series of experimental studies that address complex judgments involving integration from multiple levels of grammatical representation. They show the welcome results of the combination of theoretical research and experimental techniques when addressing such complex phenomena as NPI licensing or the emergence of scalar readings.

Zhan also provides a nice example of the usefulness of experimental methods (eye-tracking) when addressing questions such as how and when scalar and ignorance inferences are computed in disjunction phrases.

Oltra-Massuet et al. present the results of a structural priming experiment where they test two different theoretical approaches to the argument structure of (in)transitive structures. The study suggests a stronger predictive contribution of a model that supports an interpretive semantics view of syntax.

Vogelzang et al. analyze how language processing interacts with general cognitive resources by reviewing different language processing models.

Ohta et al. explore the hypothesis that topicalization and scrambling constructions are quite different in nature. They set up an experiment to assess the modular nature of these structures in Kaqchikel Mayan by identifying their main processing loci.

Finally, Gong et al. report an artificial language learning experiment studying whether hierarchies in perceptual saliency influence the learning of orders regulating adjectives of involved visual features. Their results show learning biases for orders that are congruent with the perceptual saliency hierarchy, which could contribute to the structural configuration of languages.

In a nutshell, this Research Topic offers a wide panoramic view of different stances and approaches to language and shows how the interaction of a robust theoretical apparatus, plus the application of cutting-edge data acquisition and analysis techniques can help us move forward in the understanding of a phenomenon as complex and poliedric as natural language.

## Author Contributions

All authors listed have made a substantial, direct and intellectual contribution to the work, and approved it for publication.

## Conflict of Interest

The authors declare that the research was conducted in the absence of any commercial or financial relationships that could be construed as a potential conflict of interest.

